# Prosthetics socket that incorporates an air splint system focusing on dynamic interface pressure

**DOI:** 10.1186/1475-925X-13-108

**Published:** 2014-08-01

**Authors:** Nasrul Anuar Abd Razak, Noor Azuan Abu Osman, Hossein Gholizadeh, Sadeeq Ali

**Affiliations:** 1 Department of Biomedical Engineering, Faculty of Engineering, University of Malaya, 50603 Kuala Lumpur, Malaysia

## Abstract

**Background:**

The interface pressure between the residual limb and prosthetic socket has a significant effect on an amputee’s satisfaction and comfort. This paper presents the design and performance of a new prosthetic socket that uses an air splint system.

**Methods:**

The air splint prosthetic socket system was implemented by combining the air splint with a pressure sensor that the transhumeral user controls through the use of a microcontroller. The modular construction of the system developed allows the FSR pressure sensors that are placed inside the air splint socket to determine the required size and fitting for the socket used. Fifteen transhumeral amputees participated in the study.

**Results:**

The subject’s dynamic pressure on the socket that’s applied while wearing the air splint systems was recorded using F-socket transducers and microcontroller analysis. The values collected by the F-socket sensor for the air splint prosthetic socket system were determined accordingly by comparing the dynamic pressure applied using statically socket. The pressure volume of the air splint fluctuated and was recorded at an average of 38 kPa (2.5) to 41 kPa (1.3) over three hours.

**Conclusion:**

The air splint socket might reduce the pressure within the interface of residual limb. This is particularly important during the daily life activities and may reduce the pain and discomfort at the residual limb in comparison to the static socket. The potential development of an auto-adjusted socket that uses an air splint system as the prosthetic socket will be of interest to researchers involved in rehabilitation engineering, prosthetics and orthotics.

## Background

It is generally widely known and accepted between amputees and prosthetists that a poor socket fit will entail that the stump loses volume on a daily basis [1]. The amputee’s socket interface plays a major role in defining the comfort level of the user. The method by which the socket is attached to the residual limb is extremely important [2]. Upper-extremity prostheses socket must be suspended throughout the entire range of motion as well as being able to tolerate loading during normal use [3].

From a clinical point of view, amputees need to undergo pre-post prosthetic procedures before they are provided with a permanent prosthesis socket [4]. From time to time this rehabilitation involves changes in the socket and ongoing consultation with Certify Prosthetics and Orthotics (CPOs) [4]. The amputation site has a certain volume and shape to begin with, but changes occur as the body heals and reaches a kind stability. Furthermore, the amputees may need to change the socket in response to changes in body weight or alterations to the structure of the residual limb [1,4,5].

Among the different types of prosthetic socket that can be implemented, are the harness socket [6,7], self suspending technique [8,9], and silicon liners [10], all of which utilize hybrid material engineering technologies within a prosthetic device. Socket materials and fabrication have changed over the years from leather and wood, to rigid polyester laminates, to flexible thermoplastics, and composite reinforced frames [2,11].

The static socket, which was normally fabricated and custom made by referring to guidelines from the International Committee of the Red Cross (ICRC) [12]. The ICRC preferred to develop its own technique instead of buying ready-made orthopaedic components, which are generally too expensive and unsuited to the contexts in which the organization works. The cost of the materials used in static socket devices is lower than that of the materials used in appliances assembled from commercial ready-made components [13].

The process of socket making involves; casting, polypropylene draping, assemble and shaping. The casting, rectification and alignment methods used to correspond to international prosthetic and orthotic (P&O) standards of practice and are therefore not described in the ICRC manufacturing guidelines. The measurement process involves several tools such as length calliper, universal anterior-posterior-medial-lateral calliper, standard tape, spring tape, circumferential tape, and weight scale which need to be accurately measured.

In this study, we examine the interface pressure at the socket interface. Either this finding could determine the comfort level or not is a subjective matters, but somehow, the interface pressure could related on why some amputees not comfortable and feel pain using the static socket [14]. However it could be related with the structure and weight of the residual limb. In a study by John M. Miguelez et al. [15], the authors did mention the relationship between the bones and residual contact with the socket surface whenever the load is applied. The Transradial Anatomically Contoured (TRAC) interface incorporates design elements from both the Muenster and Northwestern interfaces with more aggressive contouring of the anatomy to maximize load tolerant areas of the residual limb, as demonstrated by the radiologic analysis. In the medial/lateral plane, the interface focuses the compression anterior and slightly inferior to the epicondyles, specifically on the radial head on the lateral aspect. In addition, on the anterior/posterior plane, suspension is achieved by precisely directed compression into the cubital fold and supra-olecranon region.

The weight of the prosthetics also contributed to the impact of the pressure. Increasing the weight, which would increase the force applied, would increase the pressure. Although the study by Robert J. Dodson and Bridget Jowid [16], not formally studied, clinical observation has shown that people wearing static socket that incorporates a custom silicone interface gain greater range of motion at the elbow and wrist, report increased comfort and better tolerances of the aggressive socket design, and experience greater protection of fragile skin. A recent case study within the clinical setting highlights the positive effects of a custom silicone interface of a chronic wound and provides real observation of the benefits that the addition of this material to the prosthetic design can have on this patient population. Again, the research did mention about the comfort of socket design, but it is just in a feedback response of the user instead of experimental evidence such as interface pressure applied at the socket.

**Figure 1:**
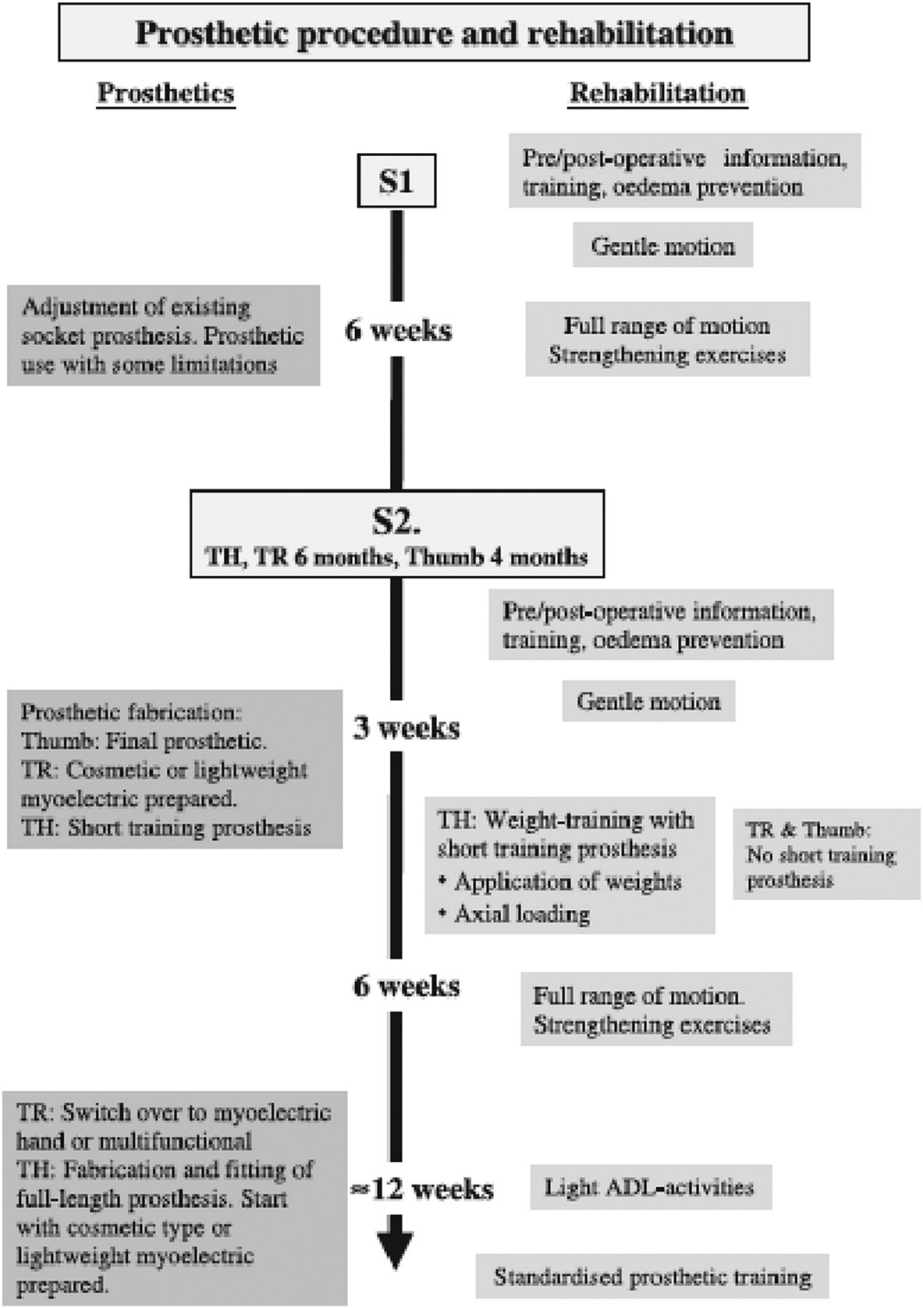
**Flowchart of pre-post prosthetic and rehabilitation procedure **[1]**.**

Although a few devices and techniques for socket interface have been discussed in existing literature [14,17], no researchers has previously examined the interface pressure of a biomechatronics system that incorporates the use of an air splint system within the amputee’s socket. This paper presents the new development of a prosthetic socket that implements an air splint system that can allow transhumeral users to make adjustments that improve the comfort level, size and fit of the socket. Firstly, the paper will discuss about condition and characteristics of commonly static socket and pre-post prosthetic socket (Figure 1). Secondly, the paper will discuss possible methods of incorporating the system between the air splint and the FSR pressure sensor, and the volume of air needed to produce by the oscillometric pump to the desired fit with the socket. Finally, the experimental performance using F-socket sensor to determine the interface pressure of the air splint socket that counters the needed for general static socket and pre-post prosthetic rehabilitation procedures.

## Methods

Pre-post prosthetic and rehabilitation procedures are common before the amputee is provided with a permanent prosthesis [1,4]. Generally, at the beginning, amputees are provided with information pertaining to the expected condition and timings of the rehabilitation procedure. After the surgery is finalized and the amputees have been forwarded to the occupational therapists, the pre-post prosthetic process begins [1,4].

The amputees are provided with a temporary prosthesis and are required to perform common simple tasks before proceeding with general tasks such as picking items up, opening a door, zipping a shirt [3,8]. This procedure is required to train the remaining muscle that is still active and allows sufficient time for the residual limb to become well shaped, which usually takes up to 6 weeks [1,4].

**Figure 2:**
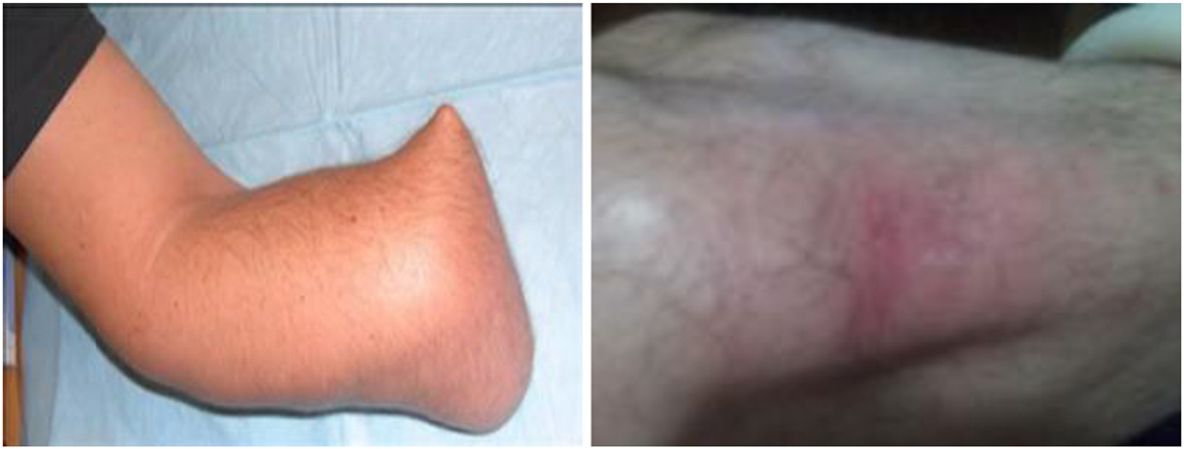
Shows different cases of residual limb condition.

**Figure 3:**
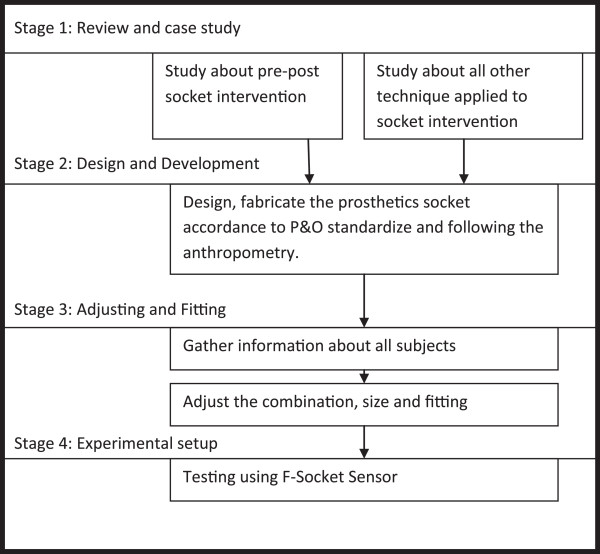
Flowchart on how the study had been overtaken.

The amputees are then provided with a permanent prosthesis and the rehabilitation of common daily task activities will continue for up to six weeks. Within the period, the amputees need to continue to perform general rehabilitation activities. It is common for amputees to revisit their therapist complaining of uncomfortable fitting within the first six to twelve months [4]. This problem usually occurs as a result of changes in the size and condition of the residual limb, which occur as a result of increases/decreases in the amputee’s body weight and height (Figure 2).In this study, the amputees and the therapist do not have to worried about the socket changing procedures. The design of air splint socket system help to auto-adjust the socket to the desired size and fitting needed by the amputees. At the same time, it reduced the consultation time for both parties and help to self-maintain the prosthetics. Figure 3 give the full flowchart on how the initial and final process in developing the air splint prostheses.

**Figure 4:**
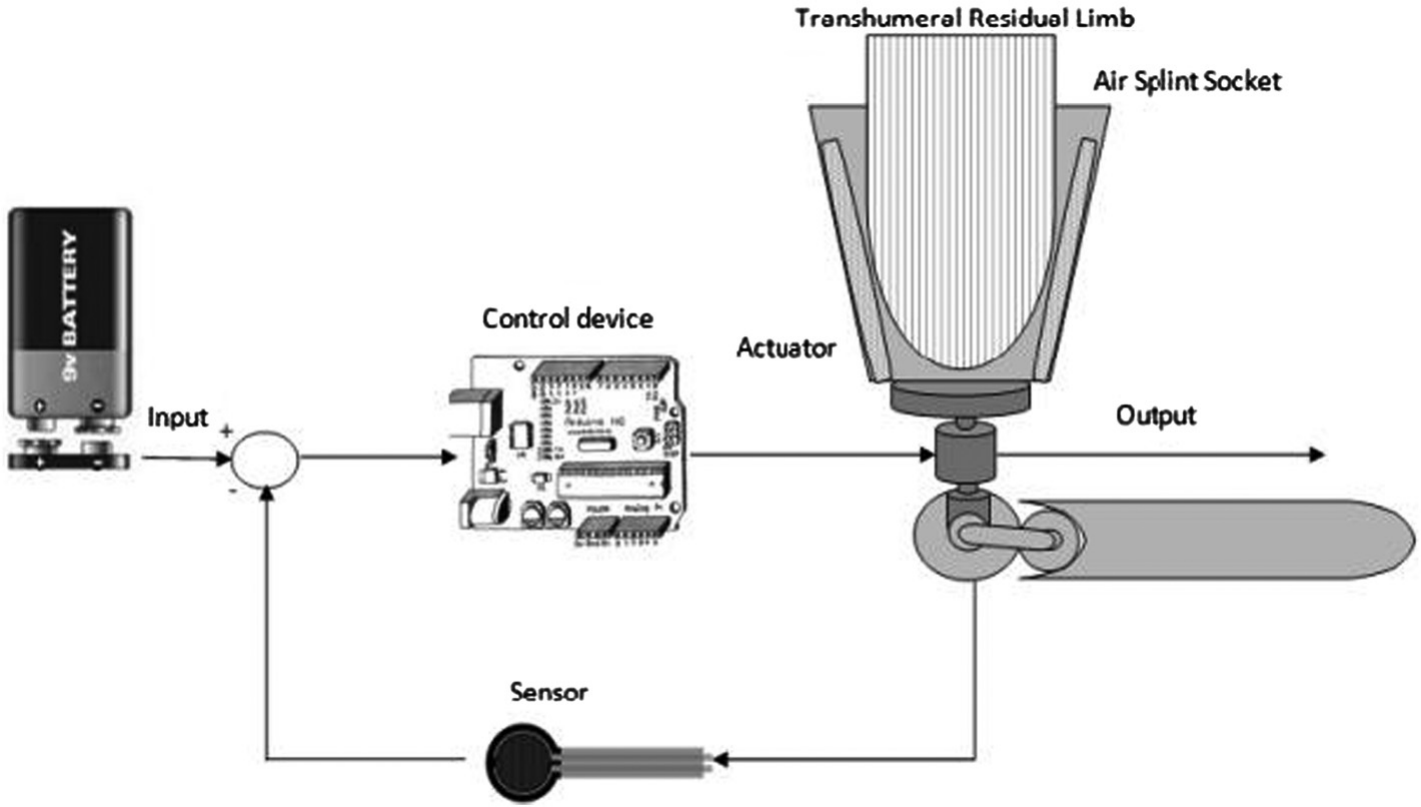
Block diagram of the air splint socket system; FSR pressure sensor used as an input, oscillometric pump used as an output, controlled by microcontroller.

**Figure 5:**
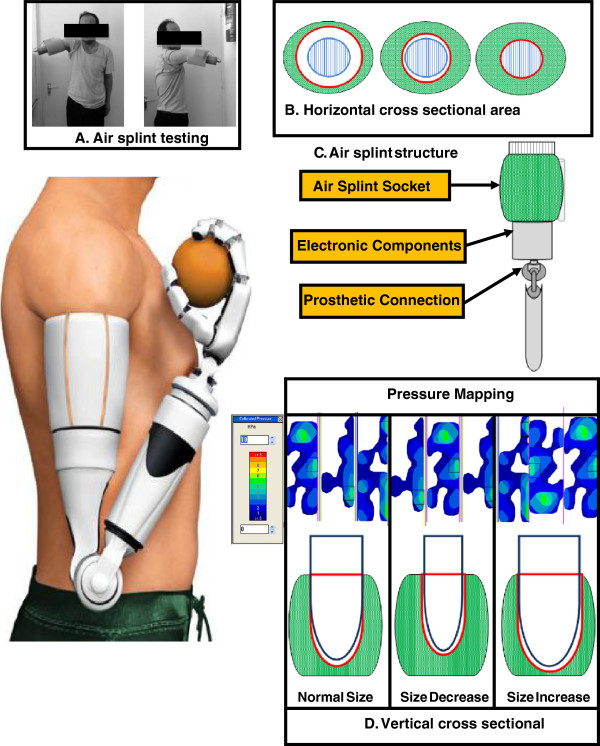
**Air splint prosthetic design and dynamic pressure mapping at the interface socket. (A)** The subject with the air splint prosthesis socket. **(B)** Horizontal cross sectional area of the transhumeral socket showing three different conditions of the air splint socket with the residual limb: (a) a minimum normal position of air splint socket, (b) the air splint socket compressed directly until the desired size and fitting, (c) the maximum air splint socket size. **(C)** Illustration of socket components. **(D)** Vertical cross sectional showing three potential designs for the air splint socket compression to the residual limb; normal, size increase and size decrease; normal position as the air pump just turned on, the air splint socket will pump up until the pressure sensor detects the desired size and required volume of 40 kPa [20,21], the air split socket will maintain the fit and size and increase and decrease the volume accordingly to maintain the required volume of 40 kPa. The pressure mapping stated how the interface pressure occur at all three condition remain maintain and the pressure was distributed constantly all over the socket interface. This condition helps to improve the proper fitting and avoid pain since the pressure applied had been distributed constantly throughout the entire socket.

The air splint socket system basically uses a FSR pressure sensor [18] (Figure 4), which is placed on the surface of the air splint socket, to transfer any pressure detection data to the microprocessor and microcontroller-based system as the input data. The FSR pressure sensor is one of the most accurate and reliable measurement tools available to determine any contact pressure between the residual limb and the socket surface [18,19]. The FSR pressure sensors use the received pressure wave to retain the input of contact within 0 kPa to 100 kPa in order to maintain the air splint system pressure accordingly to clinical principle [20,21]. If the pressure increase more than 40 kPa, the blood system will be interrupted [20,21]. A full illustration of the mechanism is shown in Figure 5. With the air splint system, the patient does not need to worry about changing the socket size and fitting, since the socket will change the size and fit accordingly within the desired contact of the residual limb.

The FSR pressure sensor that functions as the input will then send the generated data to the microcontroller system that is placed inside the upper elbow part. This part of the transhumeral also consists of an oscillometric pump that will generate the air volume that is required for the air splint or otherwise maintain it at 40 kPa [20,21]. The power supply for the system comes from 9 V batteries, which are widely available, lightweight and long lasting.

The new prosthetic component was conceived to overcome the limitations imposed at the socket. The required length and mass of the prosthetic device was designed according to Drill’s and Contini’s anthropometry theorem [22]. The development mechanism is the result of a rigorous approach, which made it possible to optimize the functionality of the socket. The articulation consisted of the air splint, which replaced the thermoplastic as the main socket part (Figure 5). The air splint incorporated a silicon liner surface in order to provide the residual limb with increased gripping force. For their own comfort and satisfaction, the amputees can use a stocking net or add another silicon liner to the residual limb; this depends on the user themselves, since the air splint socket system will adjust the size according to the required size and fitting. The electronic parts were placed at the bottom part of the air splint socket. The microcontroller, the power supply and the motor controller were placed together at a convenient joint that could be readily accessed in the event that there was a need for service or reboot. The socket was also fitted with an USB cable port that could allow the user to restart or reboot the system in the event of any problems.

The minor part of the device consisted of an oscillometric air pump. The oscillometric air pump was lightweight and integrated in the upper elbow part. The weight and size of all the devices were designed according to the exact measurements of the normal hand [22]. This was intended to counter any advance force that was applied if the amputee used a prosthetic hand. The current body-powered prostheses eliminate this factor and cause a major defect to the shoulder size [6-9].

### Ethics

The experimental protocol for this work was approved by the Ethical Community of the University Malaya Medical Centre (UMMC), Kuala Lumpur Malaysia. Written informed consent was granted by the participants from the authors for the publication. Approval ID: 829:15. One registered prosthetist fabricated all the prostheses to avoid alterations due to manufacturing, alignment and fitting. All the procedure of socket making and fitting involves the Certified Prosthetics and Orthotics (CPO) which had been recognized by International Society of Prosthetics and Orthotics (ISPO).

### Experimental setup

The experiment had been conducted to discover and compare the dynamic interface pressure using static socket and air splint socket. The experimental setup used the Tekscan F-Socket sensor (9811E). The reason of using the F-Socket was to determine the pressure surface for its flexibility, rectangular printed, and the lowest thickness. Tekscan pressure mapping systems and measurement systems were used in this experiment to provide tactile pressure mapping and force data. The pressure mapping reacts as an input was first calibrated by using the Tekscan bladder. The applied pressure will then appear in the measurement system in the computer, and all related data, such as pressure, force, and time, will be calculated. The printed circuit with a thickness just about 0.18 mm made the sensor to easily fit in the gap between the socket and the surface of the amputation level. Figure 5 shows the the F-Socket which attached to the residual limb. For the transhumeral part, only two F-Socket needed to cover almost the entire socket surface attached to the residual limb. F-Socket sensor used the F-Scan software to operate and be connected to the rear of the PC via 762 mm long cable and a cuff unit (98 mm × 64 mm × 29 mm). The cable functioned as the converter of the analogue signal to digital signal so that the data may be read by the PC. In order to have the most accurate and reliable results, some precautions were considered such as by verifying all the connections were well-organised. The cable wire was tightened to the amputee’s body part to make sure that the surface of the sensor was not disrupted.

### F-socket sensor calibrations

In this study, it was noted that every time the trial was done, the sensor was not changed, nor that a calibration was made out. For the transition between the socket and the amputation limit, the sensors that were already on the residual limb were only removed if any of the sensors was found to be defective. Before the F-Socket was fitted with the prostheses, the sensor was equilibrated and calibrated. The process of equilibrating the sensor is where the whole sensor point shares the equal amount of pressure to ensure that all 96 senses have a common output. The F-Socket was put into a pressure bladder in order to ensure that each area on the F-Socket had the similar criteria. The sensor was placed in the middle of the bladder and then was subjected to a pressure of 100 kPa by taking the specifications from the manufacturer.

### Experimental procedure

**Figure 6:**
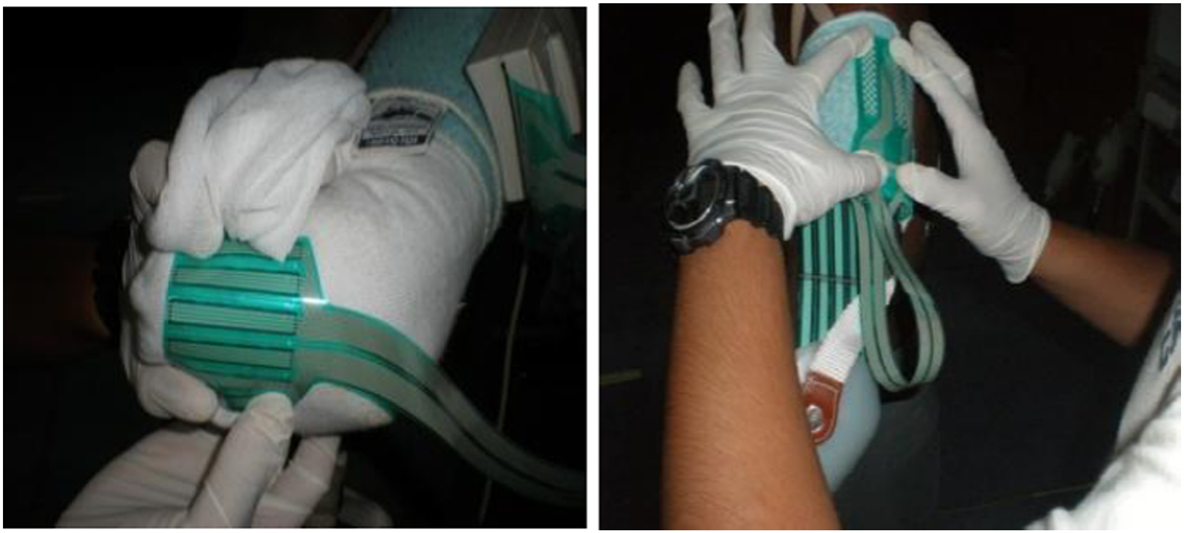
The F-socket sensor attach to the subject residual limb.

After the process was completed, the sensor was then attached to the amputee residual limb so that the position of the sensor was stable (Figure 6). Silicone liners were used for both sockets, which require no reattachment when changing the socket. The sensor was attached by using the spray adhesive, a type of strong glue. As mentioned earlier, only two sensors were required to cover the area of the residual limb. The F-Socket attached only at the part of the humerus bones that were still left. During the installation of the F-socket to the amputee’s upper elbow, the main part was to confirm that the humerus of the upper elbow was well-attached to each sensor. Since the amputation part was only 40% left, the F-Socket sensor was trimmed horizontally to reduce the length of the sensor. This step was done to accommodate the subjects with shorter limb in order to obtain a tidier sensor placement, as well as to ensure there was no overlapped sensor. After the stockinet was fully fitted into the residual limb, then the socket was fitted into the stockinet. However, the position and the liner of the sensor stability must be validated so that the data collection was not interrupted.

After the amputee was comfortable with the fitting of the socket, the F-socket sensor connects to the portable to collect some data. The value recording has a vulnerable due to the external noise that may occur. This was due to the sensitivity of the sensor and the dimensions that were physically thin but to be fitted into a small interface space. Some unwanted noises usually occurred because of the bending position for the sensor itself. There were several methods to reduce the noise distraction. The first method is by setting up the noise reduction threshold in the Tekscan’s F-Scan. The value was set up to level 3 so that any values or data below or at this level will be filtered automatically. The second method is by removing any data that were collected without applying the pressure to the sensor. When the F-Scan detected the presence of any data of unmoving pressure, the data may be diminished and the calibration of the sensor was set to zero at that level. The third way to handle this problem is by applying individual measurement to each point of the sensor. Sometimes, one of the sensors gave a high pressure and surrounded by lower pressure points. To make it stable, all of the points can detect using the F-Scan and assigned to be in a level position to each other. Therefore, the data of pressure on the interface socket can be collected precisely and correctly.

## Results

A total of fifteen transhumeral amputees (10 males, 5 females) participated in this study. All the subjects were selected from the University Malaya Medical Centre (UMMC), Kuala Lumpur and Industrial Training and Rehabilitation Centre (known as PLPP), Bangi, Malaysia. The inclusion criteria consisted of a minimum 12 cm residual limb length (from the shoulder-transhumeral bone until end of residual limb), no wound and ulcers in the residual limb, and the ability to flexion/extension shoulder without the use of assistive devices. The subjects were also considered for participation if they had used prosthesis in the last 6 months.

Even though there are many transhumeral amputees who registered to use the prosthetic hands, the majority of them had never completed the full rehabilitation training and some of them did not turn up at all. The PLPP informed that most of them did not show up because they no longer used the Body-Powered prosthetic hand due to the limitation of motion and discomfort of the prosthetic hand’s socket [3]. The subjects who participated in the study did so voluntarily and were given prior written consent letter.

The participants’ demographic information is shown in Table 1. The mean age of the subjects was (mean = 41.55, SD = 15.25). The FSR pressure sensor wave was programmed to detect any applied pressure within the range of 0–100 kPa and was placed on the surface of the air splint socket. Any pressure contact applied between the residual limb and the air splint socket could be detected by the pressure sensor. Generally, the pressure sensor detects about 0–100 kPa range according to the way in which the microcontroller has been programmed. The range increases immediately following any pressure signal and will continue to increase until the range is about 100 kPa. The air splint socket will continue to pump up until the detection reaches 100 kPa. Only then, the air splint will stop pumping and retain the pressure volume inside the air split at 40 kPa [20,21].

**Table 1:** Demographic characteristics of the participants

Gender (%)	Male	10 (66.67%)
	Female	5 (33.33%)
Weight (SD)		73.63 (12.5)
Height (SD)		170.62 (6.7)
Age (SD)		41.55 (15.25)
Side of amputation (%)	Right	10 (66.67%)
	Left	5 (33.33%)
Cause of amputation (%)	Trauma	15 (100%)

A total of fifteen transhumeral amputees (10 males, 5 females) participated in this study. The mean age of the subjects was (mean = 41.55, SD = 15.25).

It was not possible to stabilize and maintain the pressure that was required within the air splint socket during the average of the first three trials (Table 2). The pressure volume of the air splint fluctuated and was recorded at an average of 38 kPa (2.5) to 41 kPa (1.3) over three hours. The pressure of holding the socket to the residual limb should have been retained at 40 kPa by considering the skin and blood flow through the limb [20,21]. Maintaining a good fit is difficult with the total surface bearing socket because the pressure that provides a good fit causes daily volume loss in the stump [1]. For the first three hours, the contact between the skin and the socket did not stabilize (1^st^ hour; Average Pressure sensor: 101 kPa (10.3), Average Air Splint: 38 kPa (2.5), 2^nd^ hour; Average Pressure sensor: 105 kPa (14.5), Average Air Splint: 41 kPa (4.2), 3^rd^ hour; Average Pressure sensor: 107 kPa (15.6), Average Air Splint: 41 kPa (1.3)); however, after that period the pressure volume of the air splint was retained at 40 kPa per hours.

**Table 2:** Average pressure required within the air splint socket

**Time (hour)**	**Average FSR pressure sensor, kPa (SD)**	**Average air splint, kPa (SD)**
1	101 (10.3)	38 (2.5)
2	105 (14.5)	41 (4.2)
3	107 (15.6)	41 (1.3)
4	107 (23.4)	40 (2.9)
5	111 (7.8)	40 (5.8)
6	112 (5.6)	40 (3.3)
7	112 (4.7)	40 (2.8)

(N = 15). For the first three hours, the contact between the skin and the socket did not stabilize (1st hour; Average FSR Pressure sensor: 101kPa (10.3), Average Air Splint: 38 kPa (2.5), 2nd hour; Average FSR Pressure sensor: 105 kPa (14.5), Average Air Splint: 41 kPa (4.2), 3rd hour; Average FSR Pressure sensor: 107 kPa (15.6), Average Air Splint: 41 kPa (1.3); however, after that period the pressure volume of the air splint was retained at 40 kPa per hours.

## Discussion

In order for the air splint socket to maintain the pressure of 40 kPa [20,21], the pressure sensor was set up to detect any signal between the range of 0–100 kPa. If the system achieved a signal more that 100 kPa, the input data would be sent to the microcontroller to stop pumping the air split and maintain the volume of air. This sensor played a major part in defining the comfort level of the socket. As with the first three hours, the pressure sensor kept changing in the range of 101 kPa (10.3)-107 kPa (15.6. The results were directly proportional to the air splint socket results changing, as the stabilizer of the residual limb size and the socket still changed accordingly. Under the normal condition, the limb lost an average of 6.5% of its volume while doing daily life activities [1,4]. This daily volume loss occurred as a result of pressure and shear between the limb and socket [23].

The volume and condition of the residual limb plays a major part in determining the socket installation [5]. The volume of the residual limb, the bones that are left and the muscle condition play an important role in determining the type of prosthesis that should be worn by the amputee. The pre-post prosthetic user usually considers the best volume and condition of the amputated level [1]. Currently osseintegration, or the process of rehealing the bones, plays a major part if the condition of the residual limb does not meet the desired measurements to wear the prosthesis^4^. When an extremity of the body is amputated, a cascade of events takes place that impacts the already unstable system. The muscles, nerves, bones and scar tissue will not behave in the same way as they did before and even the osseintegration can not eliminate this matter [1,4,5]. After therapy, amputees might still experience pain in their residual limb as a result of neuromas and scar tissue and this can make wearing the prosthesis uncomfortable [15,16]. However, with the air splint socket system, as long as there is sufficient residual limb to wear the socket, the amputee’s comfort level will increase, regardless of the scar, muscle or bone condition.

Only a small adjustment is required via the microcontroller in the event that there are problems regards the system, otherwise the socket maintains flexibility and accurateness automatically. In cases where the residual limb reduced in size, the patients reported discomfort and swelling [6-9,15,16]. These problems would be expected in all kinds of prosthetic socket including silicon liners [10] and suction sockets [24]. These problems were corrected by using air splint socket that flexibly auto-adjusts the size of the socket according to the condition of the limb. The surface of the air splint socket incorporates silicone liners and the user can also use these together with stocking liners if they wish to eliminate the swelling problem.

In comparison to the other type of prosthesis [5-10], the harness socket can create discomfort, frustration, or difficulty in donning, restriction in range of motion [3], and contralateral brachial plexus pressure, which can potentially lead to deviate wearing the prosthetic. In externally powered prosthesis, without the socks, more aggressive anatomical contouring of the socket is needed to provide stability and control.

Other factors that can impact the socket size are control diet, hydration and activity levels [25]. Hence inappropriate diet and hydration may increase or decrease the body weight and length, which will directly change the size that is most suitable for the prosthetic. Volume loss of the residual limb ranges from 4 to 10%, with approximately 90% of this loss occurring within the first two hours of the workday [26]. This shows how the relationship between changes in socket and the rehabilitation needs to maintained on an hourly basis. Frequent changes to the socket might be too expensive to be viable and consultation with the rehabilitation unit may not be easy to maintain on a regular basis; almost 80% of prosthesis users do not attend appointments and are lost to follow-up [27]. However, the air splint socket system eliminates most of the problems that need to be considered. As long as the socket is well bonded with the amputee’s residual limb, it will maintain the desired size and fit, even when the amputee engages in daily life activities. No matter how sophisticated the device, if the patient feels uncomfortable and cannot control the placement of the terminal device, the result will be the rejection of the prosthesis.

## Conclusion

This paper presented the design and development of a new technique that uses the air splint system as a prosthetic socket replacement. Proper socket fit is crucial to the comfort of the amputee, the health of the skin and the performance of the prosthesis. The design size and measurement depends on the weight and size of each sensor, actuator and microcontroller that is incorporated in the prosthetic hand. By using a combination of a pressure sensor and an air splint system, this proposed device may overcome the problem of pre-post prosthetic rehabilitation procedure, reduce the CPOs consultation requirements and overcome the need to change the socket. The intelligence of the air splint socket system works to automatically adjust the size and fit of the socket needed. Maintaining a good fit is the main objective of the air splint socket system. However, this system cannot be applied to amputees that have a low volume, short residual limb; although this problem can be resolved by performing osseiotegration surgery on the remaining bones.

## Competing interest

The authors declare that they have no competing interest.

## Authors’ contributions

NAAR and HG designed the system and the protocol, fabricated the prostheses, conducted the experiments, collected and analysed the data, discussed the results and drafted the manuscript. NAA0 supervised the overall project, and helped in revising the manuscript. SA collected and analysed the data, discussed the results, wrote a part of the manuscript and helped in prosthetic fabrication. All the authors read reviewed the manuscript. All authors read and approved the final manuscript.
